# Digesters in traditional Persian medicine

**DOI:** 10.22088/cjim.9.1.1

**Published:** 2018

**Authors:** Zeinab Mahmoudpour, Hoda Shirafkan, Morteza Mojahedi, Narjes Gorji, Seyyed Ali Mozaffarpur

**Affiliations:** 1 Department of Persian Medicine, School of Persian Medicine, Babol University of Medical Sciences, Babol, Iran; 2 Department of Epidemiology and Biostatistics, School of Public health, Tehran University of Medical Sciences, Tehran, Iran; 3 Department of History of Medical Sciences, School of Persian Medicine, Babol University of Medical Sciences, Babol, Iran; 4 Traditional Medicine and History of Medical Sciences Research Center, Health Research Institute, Babol University of Medical Sciences, Babol, Iran; 5 Social Determent Health, Research Center, Health Research Institute, Babol University ofMedical Sciences, Babol, Iran

**Keywords:** Gastrointestinal tract, Herbal medicine, Jawarish

## Abstract

**Background:**

Functional gastrointestinal diseases are common in general populations and comprise more than 40% visits to gastroenterologists**.** Treatment options of gastrointestinal diseases have been limited. There are a few medications for functional gastrointestinal diseases and some of medications are not available in the market or in the place where the patient lives. Traditional Persian medicine (TPM) is a branch of alternative and traditional medicine based on individual viewpoint and humoral theory, focuses on lifestyle modification and uses natural products to manage the patients.

**Methods:**

In this study, a set of compound drugs known as digesters (jawarishes) and other applications are described based on main TPM text books.

**Results:**

Jawarishes have different formulations containing various medicinal herbs used for better food digestion and improved gastric functions and also used for other disorders including reinforcing the brain, heart, liver and some therapeutic approaches.

**Conclusions:**

By reviewing medieval Persian pharmaceutical manuscripts, we can conclude that many herbs are effective in different systems of the body and improve gastric functions. *Zingiber officinalis* and *Piper nigrum* are mixed together to get various formulations. The variety of jawarishes formulations and their different clinical applications can indicate continuity of their use.

Gastrointestinal diseases are common worldwide (1). Gastrointestinal disorders are about 10% of the work of hospital specialists and the prescribing costs included in the management of gastrointestinal disorders in general practice are approximately 14% of the drug budget (2). Treatment options of gastrointestinal diseases have been limited. There are a few medicines for functional gastrointestinal diseases and some of them are not available in the market or in the place where the patient lives (10). Alternative and traditional medicine methods have long history that were based on behaviors and experiments of each culture (3). Gastrointestinal (GI) diseases have importance in alternative and complementary medicine (CAM). CAM methods that have been used for GI diseases include acupuncture, electroacupuncture, herbal medicine, and behavioral therapies (1). The uses of herbal drugs in GI problems are common. It is proposed that in the general population, the use of CAM was 50.9% in patient with irritable bowel syndrome (4), 49.5% in inflammatory bowel disease (5) and 40% in pediatric patients with GI diseases (6). Traditional Persian medicine (TPM) based on individual viewpoint and humoral theory, is one of the alternative and traditional medicines that focuses on lifestyle modification and uses natural products to manage the patients (3). There is a significant role for digestive system in TPM. 

It is supposed that there is a close interaction between GI and other systems in the body. So, particular attention to gastric functions has a special role in the treatment of different kinds of diseases (7). 

Because of the importance of stomach, a set of compound drugs are introduced in TPM as digesters (jawarishes). Jawarish is an Arabic word which means "digestion of food". Jawarish*es* are herbal drugs; with hot temperament (means causing internal body heat). Jawarish is designed to improve gastrointestinal problems. In addition to their gastrointestinal effects, some of them affect other systems (8). 

In this study, we want to introduce this category of drugs known as digesters or jawarishes*. *

**Figure1:**
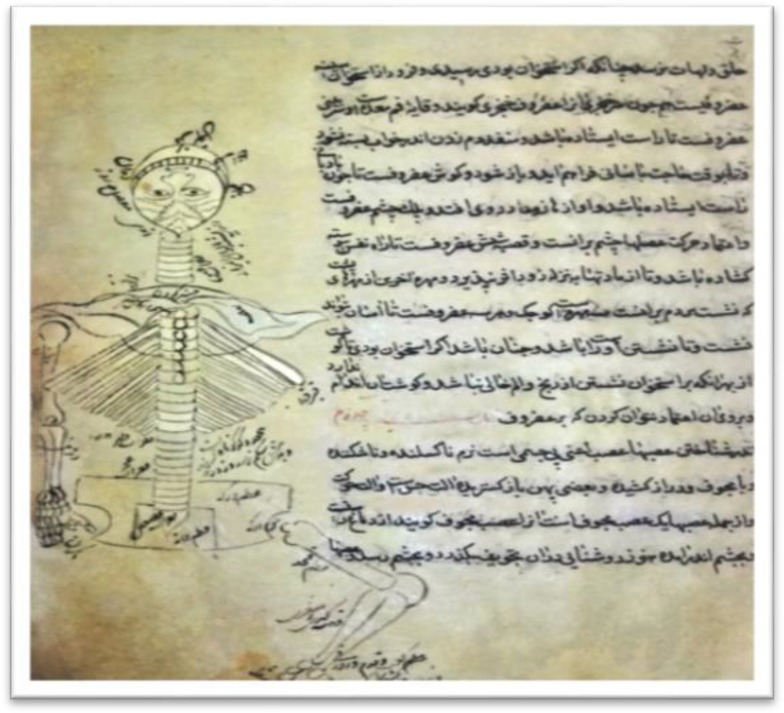
From a copy of lithograph edition of Gharabadin Shafahi of selected pictures on History of Medicine in Islam and Iran (page 43

## Methods

This review study is based on the search of the 6 major books of TPM which include “*Properties of objects”* (Khavas –al Ashyae) written by Aboo Ali, 10th century AD (9), *compound drugs of Shafahi (*Gharabadin Shafahi) by Hoseni Shafahi, 15th century AD (10), *Heaven of drugs* (Riaz al-Advieh) by Yosefi Heravi, in 15th century AD (11), *Greate* *compound drugs *(Gharabadin-e-Kabir) (8) and* Summary of wisdom* (Kholaseh al-Hekmah) by Aghili AlaviShirazi, 18th century AD (12), and *the greatest compound drugs* (Gharabadin-e-Azam) by NazemJahan, 20^th ^century AD (13). After searching the cited references with the keywords of “Javrecses”, “Hazm”,” Medeh” we categorized the results based on their proposed functions. Then their ingredients were extracted and analyzed using their prevalence data of use in different formulas. Then scientific names of materia medica were determined. Finally web- search was performed in google scholar, scientific information database (S.I.D), Scopus and PubMed to find studies about these herbs in GI functions and their probable mechanisms.

**Figure 2:**
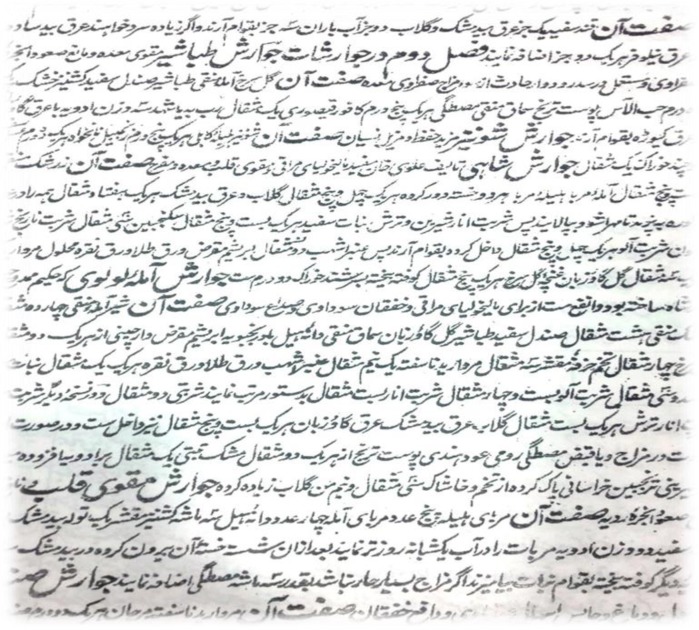
Page 19 of lithograph edition of Gharabadin Azam about digesters

## Results

As a result, jawarishes are introduced in TPM references as a group of compound drugs that can improve GI functions and can also be effective in other organs. 

Digesters in T.P.M are used in two main different indications:

1- Disorders related to GI system: Digesters are recommended for different kinds of GI disorders including reinforcement (improvement of total function including digestion) of stomach, halitosis, eructation and hiccup, bloating, constipation, some kinds of diarrhea based on T.P.M approaches, increase of appetite, colitis, hemorrhoid. *Zingiber officinalis*, *cinnamomum zeylanicum* and *Piper longum* are the most prevalent herbs in this category of drugs. Details are in table 1.

2- Disorders in other body systems: Digesters are prescribed in reinforcement of brain, heart and liver (the three main organs in the body in the viewpoint of TPM). They are also used for the management of palpitation, increase sexual desire (libido) and memory improvement (8). The herbs mostly taken in these indications are in table 2.

**Table 1:** Materia medica with the most common uses in digesters

**Type of Studies**	**Effecct**	**Scientific name**	**English name**	**Persian name**
CT	Promoting gastric emptying (14)	Zingiber Officinalis	Ginger	Zanjebil
R	Treatment of gastrointestinal disorder (15)			
CT	Treatment of functional dyspepsia (16)			
R	Antiulcer (17)			
A	Improvement in irritable bowel disorder (I.B.D)(18)			
R	Improvement in reflux (19)			
R	Achieving healthy stomach (20)			
R	Improvement in gastrointestinal tract (21)	Crocus Sativus	Saffron	Zaferan
R	Anti Helicobacter (H) pylori (22)			
A	Anti ulcer (23)			
A	Anti ulcer (24)	Syzygium Aromaticum	Clove	Mikhak
R	Prevention and reduction of colonic inflammation (25)	Piper Longum	Matico	Darfelfel
A	Therapeutic potential on amelioration of IBD (26)			
R	Improvement in constipation, diarrhea, stomachache (27)			
R	Ileum antispasmodic (28)	Valeriana Officinalis	Valeriane	Sonbolotib
R	Antigastric ulcer (29)	Cinnamomum zeylanicum	Cinnamon	Darchin
A	Gastroprotective (30)			
A	Antidiarrheal (31)			

CT: Clinical trial, R: Review article, A: Animal study

**Table 2:** Functions, number of formulas, ingredients and percentage in formulas of jawarish medicinal herbs in traditional Persian medicine references in GI system

**Functions**	**No. of formulas**	**Ingredients**	**Percent age in formulas**
Reinforcement of GI system	57	1. Zingiber officinalis 2. Cinnamomum aromaticum 3. Syziygium aromaticum	66% 54% 52%
Bloating	42	1.Zingiber officinalis 2. Piper longum 3.Piper nigrum	88% 61% 57%
Hiccup and eructation	7	1.Zingiber officinalis 2.Piper nigrum 3.Pistscia lentiscus,	71% 71% 42%

## Discussion

Digesters called jawarishes in TPM, are a group of herbal compound drugs that are prescribed to improve GI functions and other systems of the body (8). G.I effects may be attributed to promoting gastric emptying, reducing of colonic inflammation, antiemetic, antiulcer and antispasmodic effects and other system effects may be attributed to the immunomodulatory, antioxidant, anti inflammatory, prevention chronic oxidative stress damage of organs and prevention effect of ischemic injury of jawarishes. In 2012, a study by Zargaran et al. expressed some kinds of jawarishes and their important effects (32). A study claimed that the type of digesters improve nausea, vomiting, anorexia, abdominal pain, diarrhea, constipation, insomnia et c. (Arish et al. 2013) (33). A similar study demonstrated diuretic and nephroprotective effect of one type of jawarishes (Afzal et al. 2004) (34). GI is considered in TPM as an important system in the body, and it is believed it can have effect on other organs. So, to improve the function of other organs, gastrointestinal system should be observed and managed if it is necessary (7). Generally in TPM, diseases are divided in two main groups, including main or participant diseases. In a main disease the pathology is in the same organ that the signs and symptoms are presented in it. While in a participant disease, the pathology is in an organ, although the presentation is in another site. It is believed that GI can act as a source of diseases that present in other organs as a participant disease. Thus, as GI is the route of all oral drugs, they can also cause participant diseases, with regard to GI function considered important in TPM.

It has been proposed in recent studies that most of the materia medica used as principles of digesters have effect on GI. *Zingiber officinalis* is a common herb and the most frequently used herb in formulas of digesters. Combinations of herbs in digesters are purposeful. It is assumed that there is one or more ingredients in each compound drug that cause the main effect. For example, it is conceived that some ingredients in combinations act as reducers of the major side effects. There may be some ingredients in the formula that enhance penetration, and help increase drug potency with a lesser amount of main herbs. By reviewing the pharmaceutical manuscripts of mediecal persia, we can conclude that jawarish ingredients have hot temperament (means causing internal body heat) and many effective herbs on systems of the body improve gastric functions. *Zingiber officinalis* and *Piper nigrum* are mixed together in various formulations. The variety of jawarish formulations and their different clinical applications can indicate continuity of their use. In Qarabadin books, traditional kinds of digesters have been defined and categorized. Most of them improve the GI systems and some of them have other effects, too. In recent years attention to traditional formulas has increased and many studies have been and are being conducted all over the world. These formulations are considered for special diseases and patients with the least side effects by scientists. This adjuvant therapy should be selected for clinical trial studies. If the same results are conformed, it should be implemented for the prevention and treatment of different gastrointestinal problems and other complications.
